# Personalized molecular modeling for pinpointing associations of protein dysfunction and variants associated with hereditary cancer syndromes

**DOI:** 10.1002/mgg3.447

**Published:** 2018-07-24

**Authors:** Sarah Macklin, Ahmed Mohammed, Jessica Jackson, Stephanie L. Hines, Paldeep S. Atwal, Thomas Caulfield

**Affiliations:** ^1^ Department of Clinical Genomics Mayo Clinic Jacksonville Florida; ^2^ Department of Medicine Division of Diagnostic & Consultative Medicine Mayo Clinic Jacksonville Florida; ^3^ Center for Individualized Medicine Mayo Clinic Jacksonville Florida; ^4^ The Atwal Clinic Jacksonville Florida; ^5^ Department of Neuroscience Mayo Clinic Jacksonville Florida

**Keywords:** computational screening, hereditary cancer, protein modeling, variant classification

## Abstract

**Background:**

Although the process of reclassification of a variant of uncertain significance can be complex, they are commonly detected through molecular testing. It often takes years before enough clinical data are acquired, and it can be costly and time‐consuming to perform functional analysis of a single variant. It is important that other tools are developed to aid in clarifying how a specific genetic variant impacts a protein's function, and ultimately the health of the patient.

**Methods:**

Two more newly characterized, suspected pathogenic variants in *NBN* and *PTEN* were analyzed through personalized protein modeling. Comparisons between the wild‐type and altered protein were studied using simulations, genomic exome analysis, and clinic study.

**Results:**

Modeling of the new *NBN* and *PTEN* protein structures suggested loss of essential domains important for normal enzymatic function for these personalized genomic examples which matched the clinical findings.

**Conclusion:**

The defects detected through modeling were consistent with the expected clinical effect. Personalized protein modeling is another tool for determination of correct variant classification, which can become further useful through construction of deposition archive.

## 1 INTRODUCTION

Of the numerous individuals who complete clinical genetic testing, a significant portion will receive an inconclusive result (Altschul et al., 1997). Variants of uncertain significance are genetic alterations with an unclear impact on gene and downstream protein function. One‐third of individuals tested with a 25 gene hereditary cancer panel will receive at least one Variant(s) of Unknown Significance (VUS) (Altschul et al., 1997), and the number of uncertain results discovered generally increases with number of genes tested (Amendola et al., 2016). It is not always clear though whether a specific variant should be classified as (likely) pathogenic, uncertain significance or (likely) benign. Guidelines have been put forward by the American College of Medical Genetics and Genomics and the Association for Molecular Pathology to aid in the classification of variants (Caulfield, 2011), but there can still be disagreement amongst laboratories (Caulfield & Devkota, 2012). It often takes a long period of time for enough clinical data to accumulate to allow for confident reclassification of a VUS to pathogenic or benign (Caulfield, Devkota, & Rollins, 2011; Caulfield & Medina‐Franco, 2011), and functional studies can be costly and time consuming. Computational and predictive data, like protein modeling, can aid geneticists in this task. Personalized molecular modeling and structure‐based analytics from comparison of VUS and wild‐type represent an expanding toolkit at the physicians practice.

## 2 CASE PRESENTATION

The first variant, previously discussed in a case report (Garcia, 2014), presents as a frameshift variant of *NBN* (OMIM No. 602667), c.698_701delAACA, which was classified by the original testing laboratory as pathogenic. The patient had a history of contralateral breast cancers, with a diagnosis of left breast ductal carcinoma in situ at age 62 and right breast invasive ductal carcinoma at age 64. Her medical history was also significant for melanoma on the lateral corner of the left eyelid, melanoma on the left posterior calf at age 40 and squamous cell carcinoma of the skin at age 65. Her family history included her mother with breast cancer at age 69, a brother with renal cancer at age 59, a sister with melanoma in her 50's and a maternal half‐brother with small bowel lymphoma. While no heterozygous individuals had been reported previously, this *NBN* variant had been seen previously in homozygous individuals with Nijmegen breakage syndrome 1 (Gass et al., 2017).

The second genetic change studied was a pathogenic deletion of exon 8 in the *PTEN* gene (OMIM No. 601728). The patient presented with a recent diagnosis of endometrioid cancer of the ovaries with synchronous endometrial intraepithelial neoplasia and an endocervical adenomcarcinoma at age 37. Her past medical history included a partial thyroidectomy for premalignant lesion at age 15, a thyroidectomy for premalignant lesion at age 34, and a premalignant melanoma at age 31. She also had a history of multiple colon polyps, pathology unavailable, discovered around age 22 and 35, as well as, a ganglioneuroma that was discovered at her most recent colonoscopy at age 37. She was adopted, and her family history was unknown. Genetic testing identified a deletion of exon 8 in *PTEN* which was originally classified as a VUS and then upgraded to pathogenic by the testing laboratory some weeks later. Physical examination identified macrocephaly, consistent with *PTEN*‐related disease. She was referred to dermatology and found to have a sclerotic fibroma/storiform collagenoma. Her son subsequently tested positive for the deletion. He had no history of cancers or tumors, but did have a history of macrocephaly, premature birth at 29 weeks and early developmental delay that had largely resolved. As this *PTEN* variant and the pathogenic *NBN* variant were less well characterized with regards to hereditary cancer syndromes, they were good examples to test with protein modeling.

## 3 MATERIALS AND METHODS

Computer assisted modeling was completed using a sequence of human protein nibrin (NBN) taken from the NCBI Reference Accession Sequence: NP_002476: version NP_002476.2 (GenBank: AF069291.1), which is encoded for a 754 amino acid sequence. The required PTEN sequence was also taken from the NCBI Reference Accession Sequence: NM_000314: version NM_000314.6 (GenBank: AF067844.1), which encodes a 403 amino acid sequence.

Monte Carlo (MC) simulations were performed on the altered proteins to observe local and regional changes for 754 amino acids for the NBN protein and 403 amino acids for the PTEN protein. For the NBN protein, frameshift begins at K233, and subsequently leads to a deletion construct, such that we have S233, I234, R235, N236 and then deletion of the remaining 518 residues dubbed “K233SIRN(X)”. The p.del(D268‐K342) variant was introduced to the PTEN protein. The X‐ray refinement for Monte Carlo was built using the YASARA SSP/PSSM method (Hooft, Sander, Scharf, & Vriend, 1996; Hooft, Vriend, Sander, & Abola, 1996; Humphrey, Dalke, & Schulten, 1996; King & Sternberg, 1996; Krieger et al., 2009; Laskowski, MacArthur, Moss, & Thornton, 1993). Relaxation of the structure was completed via the YASARA/Amber force field using YASARA knowledge‐based refinement protocol (Lopez‐Vallejo et al., 2011). Schrodinger's LC‐MOD Monte Carlo‐based module and minimization finalized the models, generating initial model and mutant protein (Hooft, Sander, Scharf, & Vriend, 1996; Hooft, Vriend, Sander, & Abola, 1996; Humphrey et al., 1996; Krieger et al., 2009; Murray, 2011). Conformational sampling was done through Monte Carlo dynamics searching (LCMOD‐MC) (Qiu & Elber, 2006; Rehm et al., 2013; Reumers et al., 2005; Richards et al., 2015). The systems then proceeded to the MC search criteria (Qiu & Elber, 2006; Rehm et al., 2013; Reumers et al., 2005; Richards et al., 2015). MC helped study any VUS and possible effects on DNA binding or processing.

The full‐length structure for the wild‐type (WT) NBN protein was 754 amino acids (11,919 atoms) and 236 amino acids (3,710 atoms) for the K233SIRN(X) variant. For the PTEN protein, dimeric complexes of 161 amino acids (5,017 atoms) of the full‐length wild‐type model and 91 amino acids (2,970 atoms) for the p.del(D268‐K342) variant were reviewed.

## 4 RESULTS

Both of these proteins, NBN and PTEN, form internal domain interactions that are important to function, and the structural modeling drawn from X‐ray structural datasets demonstrated a strong effect from the respective variants.

**Figure 1:**
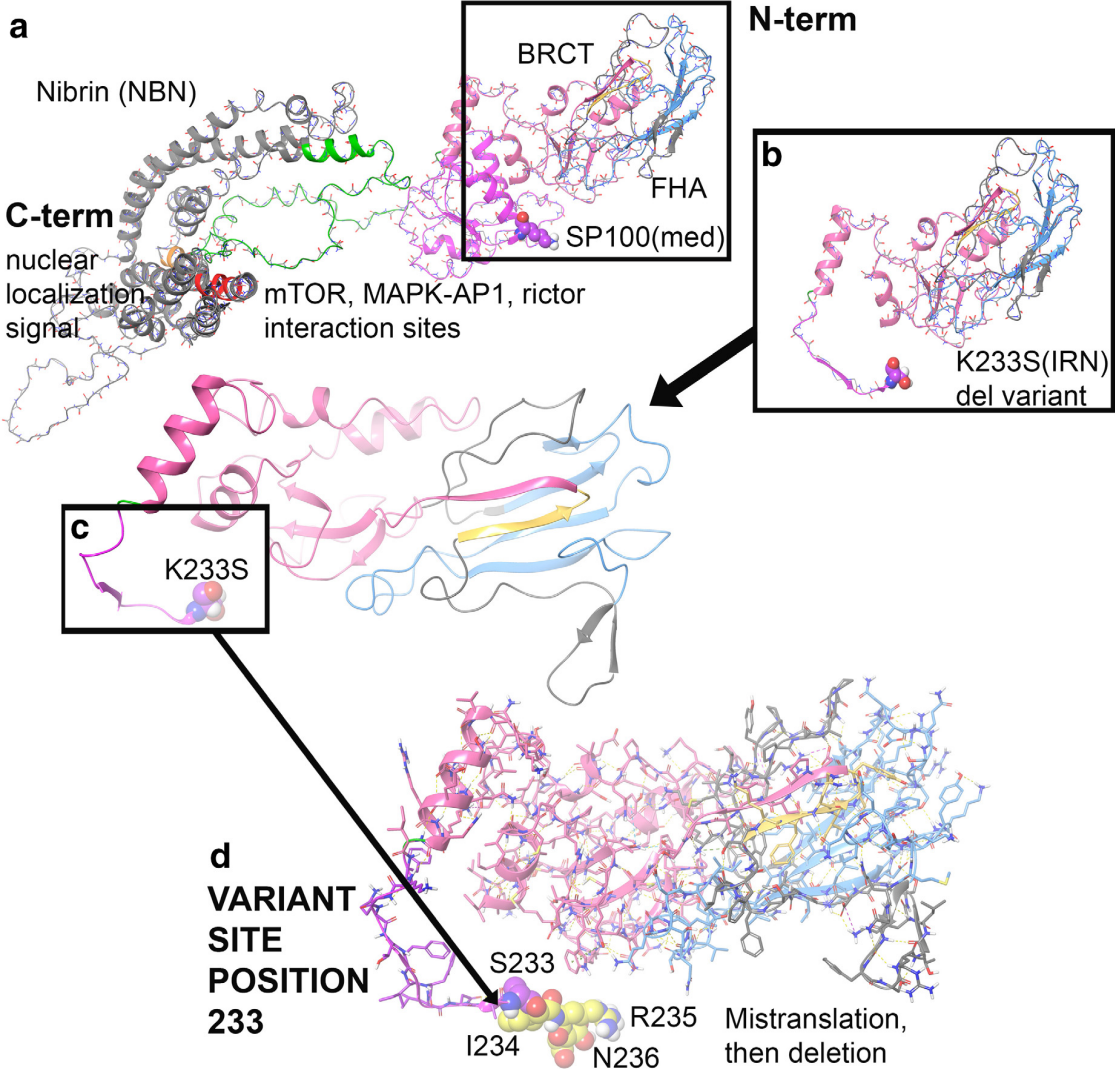
NBN molecular model for full‐length human sequence consisting of 754 amino acids and the truncation variant K233SIRN(X). (a) Full‐length structure for NBN with interaction between the domains critical for function is shown. (b) Truncation structure for NBN with S233, missense mutations I234‐N236, and deletion of 237‐754. (c) Zoom to p.K233S site with ribbons only and position 233 in VdW. (d) K233SIRN(X) is shown

The wild‐type was compared to the NBN truncation variant K233SIRN(X), and the stability of the object from energetic calculations for ΔG per amino acid is lower for the K233SIRN(X) variant. WT and K233SIRN(X) were 580.98 and 146.79 kcal/mol*Å2 respectively (Caulfield, 2011; Caulfield & Devkota, 2012; Caulfield & Medina‐Franco, 2011; Lopez‐Vallejo et al., 2011; Reumers et al., 2005; Schymkowitz et al., 2005).^24^ Per amino acid, they measured 0.77 kcal/mol*Å2 for the WT and 0.62 kcal/mol*Å2 for K233SIRN(X). Interestingly, the K233SIRN(X) protein fragment had better total object stability when not examined at a per amino acid distribution of energies. Nibrin is involved in DNA damage repair and aids with histone‐related protein recruitment; better object stability may make the K233SIRN(X) variant better for N‐terminus binding over the WT protein. However, the huge deletion of 69.1% of the NBN protein for K233SIRN(X) results in massive defect in the protein's function, which eliminates binding partners. The actual residue mutation, p.K233S, induces a +0.28808 kcal/mol*Å2 change in free energy, which is disruptive to the local region. However, given the additional missense mutations from I234‐N236, functional impact is much greater. The molecular model for the full structure and its deletion form are given (Figure 1b–d) (Caulfield, 2011; Caulfield & Devkota, 2012; Caulfield & Medina‐Franco, 2011; Caulfield et al., 2011; Lopez‐Vallejo et al., 2011).^24‐31^

Examination of the domain regions lost due to the NBN truncation consists of residues within the SP binding region (residues E111‐H238) and mTOR, MAPKAP1, RICTOR binding sites (residues I221‐T402) (Figure 1a). Nuclear localization signal is lost (P461‐E467) and the EEXXXDDL motif is gone (E736‐L743) (Figure 1a). The electrostatic distribution map (ESM) and portions for the deleted regions were also determined (Supporting information Figure S1).

**Figure 2:**
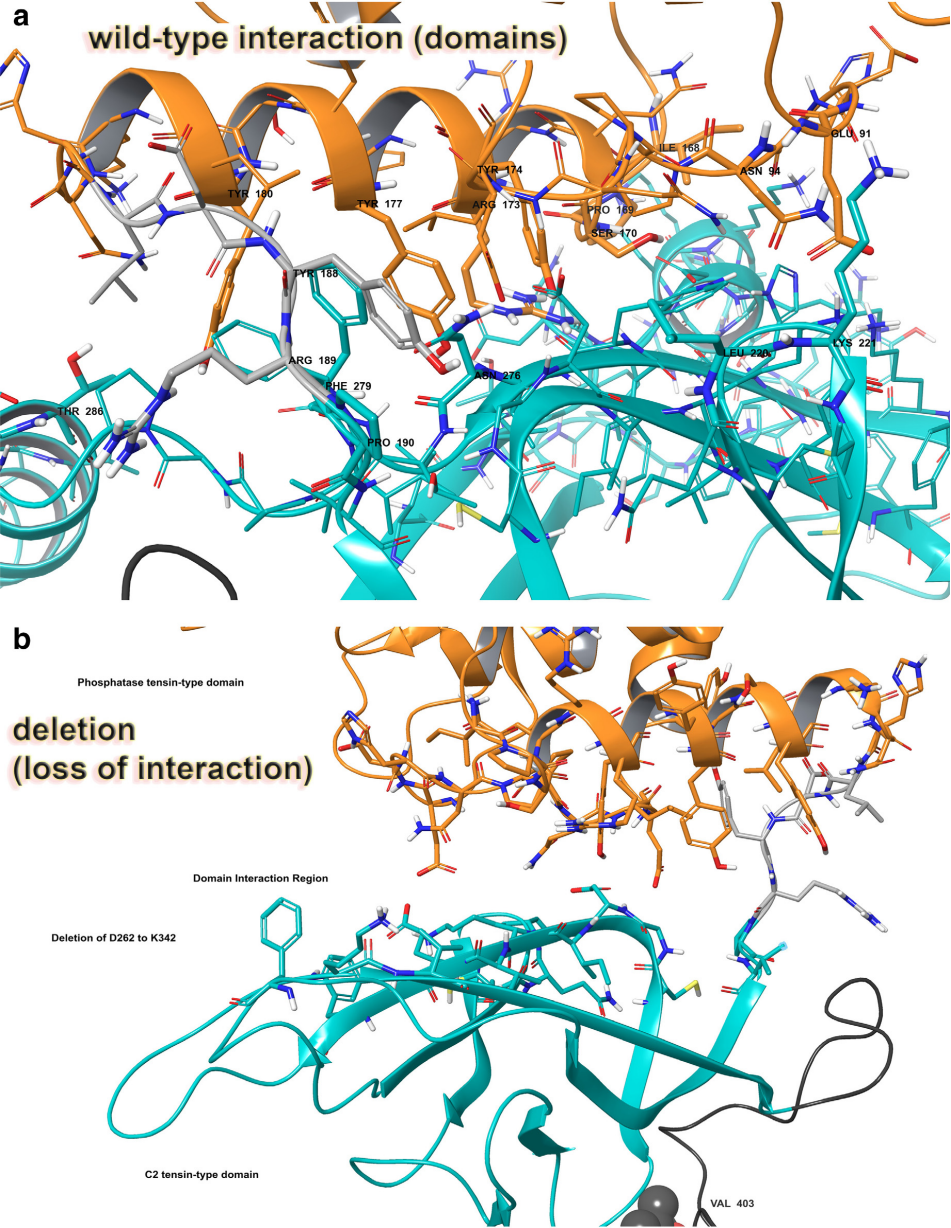
PTEN molecular model for full‐length human sequence consisting of 403 amino acids and the deletion variant p.del(D268‐K342). (a) Full‐length model for PTEN with interaction between the critical domains shown. (b) Deletion construct for PTEN with residues 262 through 342 absent in this translated construct

For WT versus the PTEN deletion p.del(D268‐K342), the stability of the object from energetic calculations for ΔG per amino acid to is higher for the deletion. WT measured 164.68 kcal/mol*Å2, and p.del(D268‐K342) measured 214.53 kcal/mol*Å2 (Qiu & Elber, 2006; Rehm et al., 2013; Reumers et al., 2005; Schymkowitz et al., 2005; Tung et al., 2016; Varon et al., 1998). This object stability does indicate the new structure is deleterious to function based on visual inspection of domain interactions, but also on the energetics output provided by the foldx algorithm (Varon et al., 1998). However, the large deletion of 18.4% of the protein with p.del(D268‐K342) results in a rather impressive defect in the protein's function due to location at the domain interface (Figure 2a–b) (Qiu & Elber, 2006; Rehm et al., 2013; Reumers et al., 2005; Richards et al., 2015; Schymkowitz et al., 2005; Varon et al., 1998).

**Figure 3:**
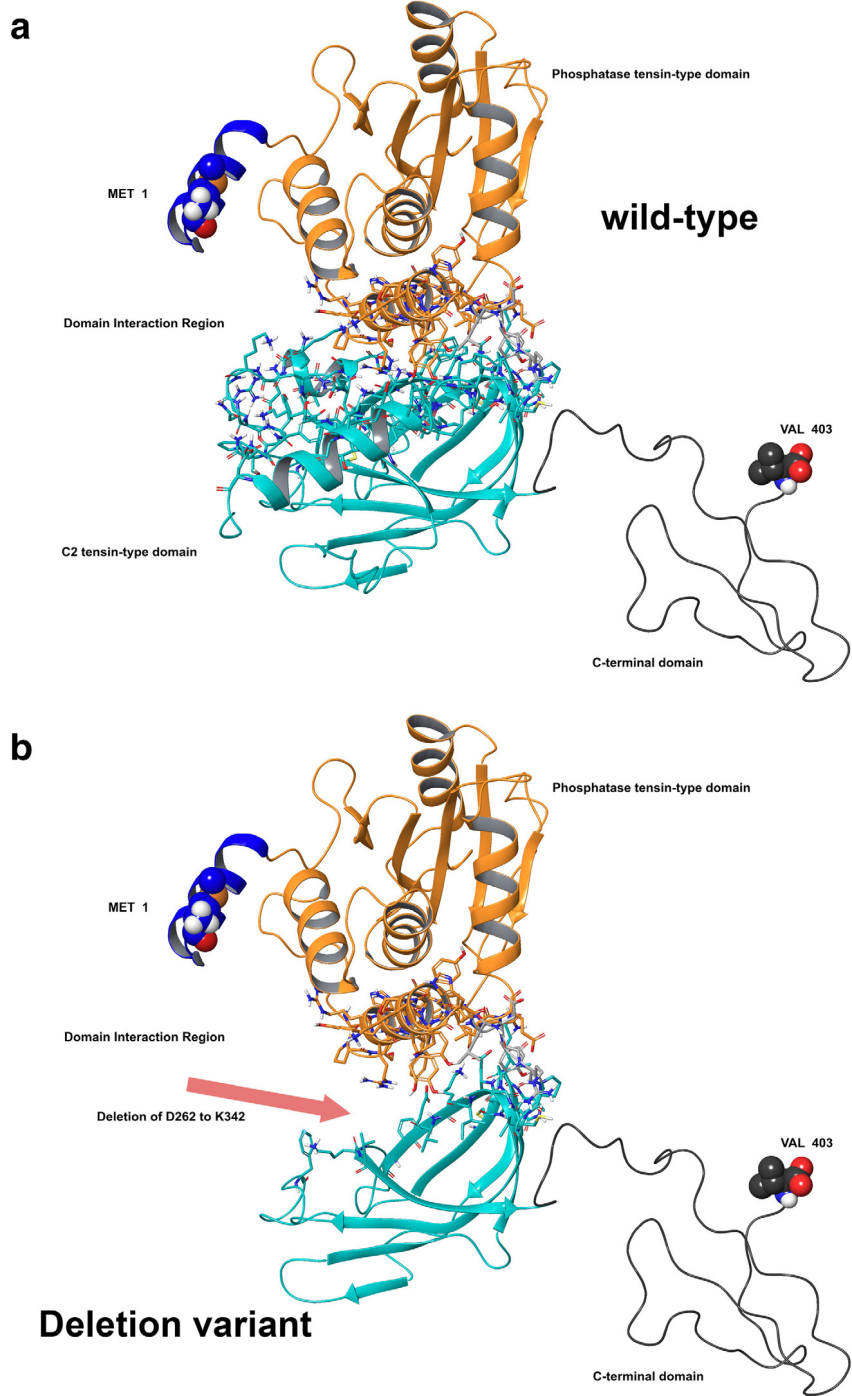
PTEN domain interactions: wild‐type versus deletion variant p.del(D268‐K342). (a) Critically important residues between the domains for function are shown. Interaction residues are labeled. (b) Deletion construct for PTEN with residues 262 through 342 absent leaving a large gap between the domains, effectively reducing any domain‐domain interactions, lowering enzyme function. Rendering and coloring is same as in PTEN Figure 2

Examination of the domain interaction zone in that region of PTEN, which consists of residues E91, N94, I168, P169, S170, R173, Y174, Y177, Y180, Y188, R189, P190, L220, K221, N276, F279, and T286, identifies helix‐helix and helix‐beta sheet interactions (Figure 2a). Specific hydrophobic interactions between the helices involve I168, P169, P190, L220, N276, and F279, and hydrogen bonding (Figure 3a). We also examined the ESM for PTEN (Supporting information Figure S1).

## 5 DISCUSSION

As it is justifiable to immediately think that frameshift and other truncating variants can cause nonsense mediated decay and no protein product, previous studies have shown that this is not always the case. Tanzarella et al. 2003 in his paper described two *NBN* variants (835del4 and 900del25), which were translated into a form of Nibrin with smaller molecular weight (Tanzanella, 2003 #2). Using established techniques, the energetics data and modeling reveal conclusive defects in the NBN protein and the PTEN protein (Qiu & Elber, 2006; Rehm et al., 2013; Reumers et al., 2005; Richards et al., 2015; Schymkowitz et al., 2005; Tung et al., 2016; Varon et al., 1998). The models for the NBN structure and PTEN structure show loss of essential domains interactions for proper function. In both cases, the energetic calculations structure show in great detail how the deletion construct has been perturbed from the wild‐type protein in a manner that may put the native protein at a disadvantage when competing for N‐terminus binding partners; thus promoting loss of function or decreased activity. The structural models of these two variants concur with previous clinical data. We are continuing to build a database for all *NBN* and *PTEN* VUSs for future use to the clinical community as an online publicly available resource, which will become available after we have added sufficient number of cases. Protein modeling should be considered as another tool in the difficult task of variant classification.

## CONFLICT OF INTEREST

The authors have no conflicts of interest to declare**.**
