# Supplementary figures and images for: Personalized molecular modeling for pinpointing associations of protein dysfunction and variants associated with hereditary cancer syndromes
Source: Mol Genet Genomic Med. 2018 Jul 24;6(5):805–10. doi: 10.1002/mgg3.447 (PMC6160717; doi:10.1002/mgg3.447)

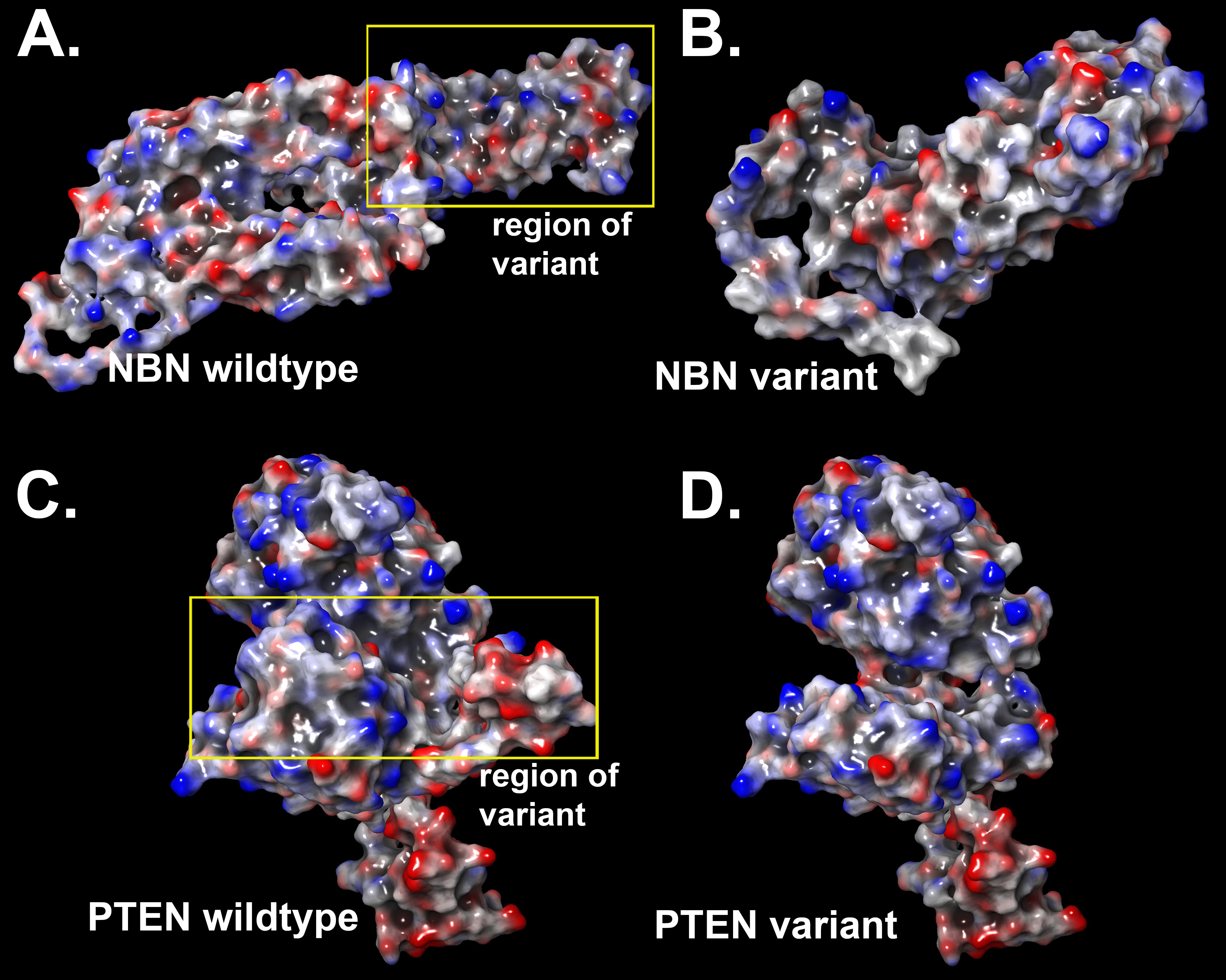

Supplement: Supplementary file 1 [file MGG3-6-805-s001.jpg]
